# Idiopathic spontaneous intraperitoneal haemorrhage: A near fatal presentation of acute abdomen requiring prompt diagnosis

**DOI:** 10.1016/j.ijscr.2023.108650

**Published:** 2023-08-10

**Authors:** Danniel Badri, Callie Killoran, Ratna Aseervatham

**Affiliations:** a Department of Surgery, Sunshine Coast University Hospital, 6 Doherty Street, Birtinya, Queensland, 4575, Australia; b University of Queensland, Faculty of Medicine, 288 Herston Road, The University of Queensland, Brisbane, Queensland, 4006, Australia; c Griffith University, School of Medicine and Dentistry, Parklands Drive, Southport, Gold Coast Campus Griffith University, Queensland, 4222, Australia

**Keywords:** Idiopathic spontaneous intraperitoneal haemorrhage, Abdominal apoplexy, Post-partum, Middle colic artery rupture, Pseudoaneurysm, Case report

## Abstract

**Introduction and importance:**

Idiopathic spontaneous intraperitoneal haemorrhage is a rare and life-threatening condition that results from non-traumatic visceral artery rupture in the latter half of pregnancy and within the postpartum period [1–3].

**Case presentation:**

A 32 -year-old woman presented to emergency department, 14 weeks post-partum, with sharp left sided abdominal pain, nausea, and vomiting. Initial computed tomography (CT) was suggestive of non-specific colitis from transverse to descending colon of unclear cause. Six hours into admission she became haemodynamically unstable with abdominal peritonism resulting in emergency laparotomy. Intra-operative findings showed large volume haemoperitoneum with an active bleed from the middle colic artery.

**Clinical discussion:**

Symptoms and clinical presentation of Idiopathic spontaneous intraperitoneal haemorrhage is variable and ranges from vague abdominal pain to haemorrhagic shock. A latent period of several hours may be followed by a rapid progression of symptoms owing to rapidity of extravasation[3]. Pathogenesis has been suggested to arise from the increased physiologic demands during the intrapartum period, wherein repeated distension of vessels and increased tortuosity leads to a predisposition for rupture [4].

**Conclusion:**

Diagnosis of Idiopathic spontaneous intraperitoneal haemorrhage is difficult but should be a differential in those who are post-partum presenting with abdominal pain. Patients should be assessed with CT angiography and treatment focused around aggressive resuscitation, surgical exploration, and ligation [3].

## Highlights

• Idiopathic spontaneous intraperitoneal haemorrhage is a rare and life-threatening condition.
• Pathogenesis is of non-traumatic visceral artery rupture, with 30 % of cases having no identifiable cause.
• Unprovoked peritoneal haemorrhage can occur within latter half of pregnancy and the postpartum period.
• Treatment is centred around aggressive resuscitation, surgical exploration, and ligation.

## 1 Introduction

Idiopathic spontaneous intraperitoneal haemorrhage, historically known as abdominal apoplexy, is a rare and life-threatening condition [1,2]. The pathogenesis includes non-traumatic visceral artery rupture, generally from pseudoaneurysm or aneurysm, with 30 % of cases having no identifiable cause [1,3]. Spontaneous arterial rupture is not necessarily preceded by an aneurysmal stage [1]. There is a 2–3:1 male:female predominance [1,3].

Unprovoked peritoneal haemorrhage is reported as an entity in the latter half of pregnancy and within the postpartum period [2]. Whilst relatively rare in this context, an index of suspicion should exist in a patient presenting with unexplained abdominal pain and haemodynamic instability [2,4]. A postulated pathogenesis is of repeated distension of vessels during pregnancy, in the presence of additional vascular defects, predisposing to rupture [4].

Due to recent advances in anaesthetic, resuscitative, and surgical technique, the mortality rate has been ameliorated to approximately 3.6 % [2]. Reported cases in literature describe 19 % of cases as intrapartum, 61 % pre-labour, and 21 % in the puerperal phase [2]. Presented is a case of a 32-year-old-woman who presented with idiopathic spontaneous intraperitoneal haemorrhage, with sudden hypovolaemic shock necessitating operative intervention for a middle colic artery pseudoaneurysm. This case is reported in line with the SCARE criteria and informed consent was obtained from patient for publication of case report [5].

## 2 Presentation of case

A 32-year-old woman, 14 weeks postpartum, presented to an emergency department after 3 days of sharp left sided abdominal pain, nausea, and vomiting. The patient had no other past medical or surgical history, was G6P6, and had otherwise experienced an uneventful pregnancy.

Initial examination revealed normal vital signs, left abdominal palpation tenderness, and no signs of peritonism. Blood work demonstrated a white cell count of 13.9 × 10^9^/L and a haemoglobin of 150 × 10^9^/L. Initial computed tomography showed a segmental bowel wall thickening from transverse to descending colon, suggestive of colitis.

Six hours into admission, the patient rapidly deteriorated into hypovolaemic shock, with hypotension, tachycardia of 150 bpm, cool peripheries, localised left sided peritonism, and a lactic acidosis (lactate 8.8 mmol/L, pH 7.18). Unsuccessful fluid resuscitation, increasing abdominal pain and a repeat haemoglobin of 121 × 10^9^/L, resulted in the decision to proceed to laparotomy.

**Fig. 1:**
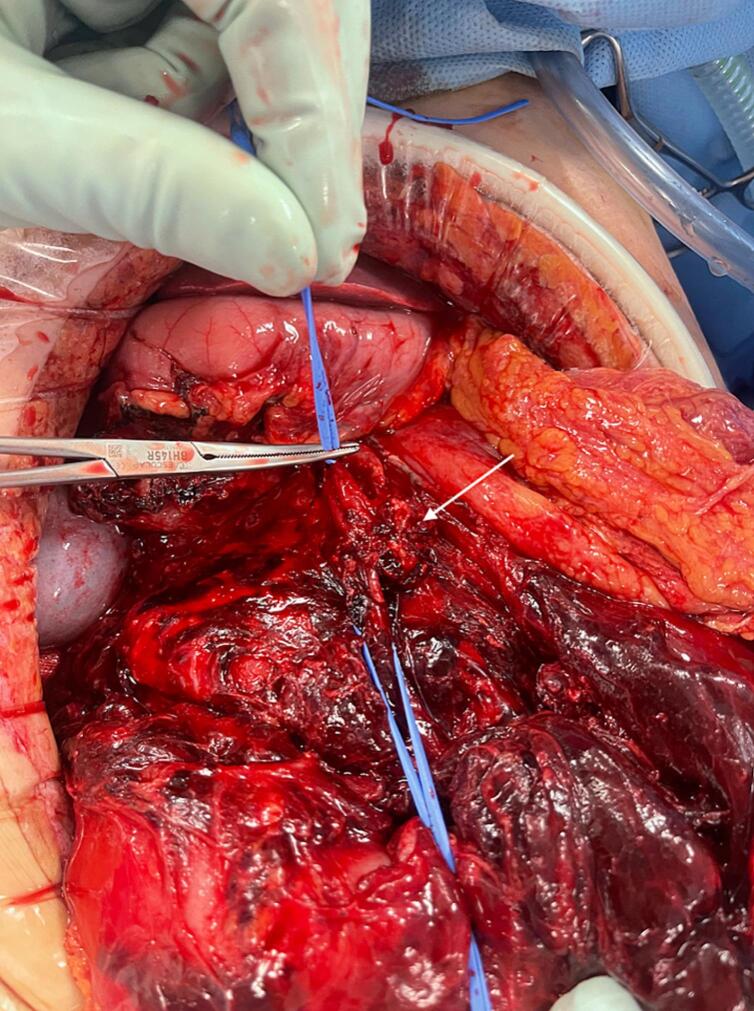
Intraoperative image demonstrating previously ligated irregular branch (arrow) within fibrin and haematoma, originating from middle colic artery (artery and vein demonstrated proximally and distally by blue loops).

Early instability noted under anaesthesia necessitated supra-coeliac aortic clamping to allow for transfusion and inotrope commencement. Intraoperative findings were of large volume haemoperitoneum due to active haemorrhage from a branch of the middle colic artery (Fig. 1). The vessel was ligated and the abdomen left open for a re-look procedure once stable.

**Fig. 2:**
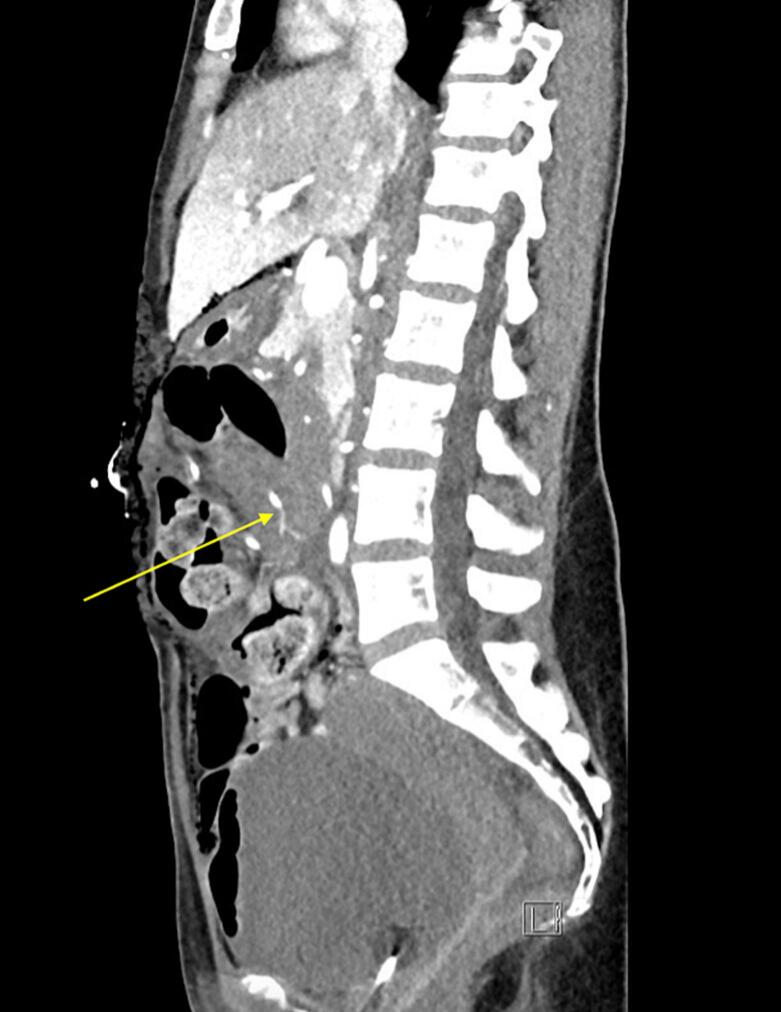
Computed tomography angiography demonstrating 5 mm pseudoaneurysm from irregular arterial branch of middle colic artery (arrow), with associated surrounding haemoperitoneum.

**Fig. 3:**
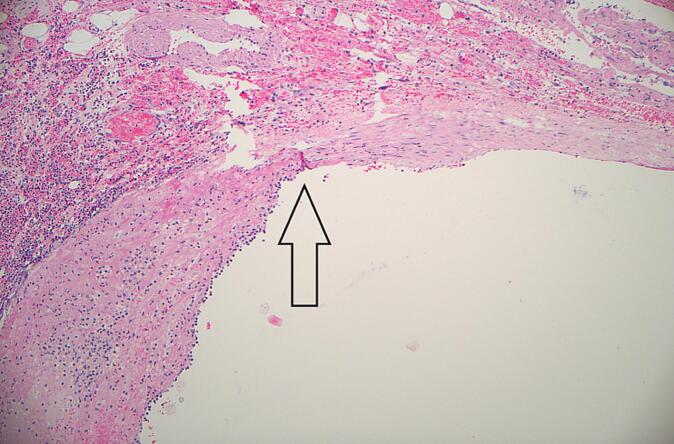
Histopathology (H&E stain) of resected irregular branch of middle colic artery (arterial wall). Demonstrated is transition (arrow) from viable smooth muscle (right of arrow) to area of disruption and inflammation (left of arrow).

A CT angiogram performed the next day demonstrated a 5 mm pseudoaneurysm in continuation with an irregular branch of the middle colic artery, potentially representing the site of previous haemorrhage (Fig. 2). Re-look laparotomy at a 36 h interval allowed for formal ligation and resection of the middle colic artery and vein proximal to the previously ligated branch. Histology revealed a resected segment of arterial wall, with haemorrhage, fibrin thrombus, patchy acute inflammatory exudate, and focal necrosis (Fig. 3). The patient had an uneventful recovery and will be followed up with surveillance imaging to assess for further vascular abnormalities.

## 3 Discussion

Idiopathic spontaneous intraperitoneal haemorrhage is a rare and life-threatening condition that refers to the spontaneous rupture of the abdominal splanchnic vasculature [6]. It is a term used to describe non-traumatic spontaneous and atypical intraperitoneal bleeding [7]. The main suspected cause is from abdominal aneyrysm with 60 % as a result from the splenic artery [1,7]. Aneurysms of the colic arteries are scarce (less than 0.3 % of all superior mesenteric aneurysms) and there are accordingly few reported cases of middle colic artery rupture [8]. Risk factors include hypertension and arteriosclerosis, however, is linked to older population base with no known specific risk factors in pregnancy beside pregnancy itself [1,7,9]. It is suggested that the increased physiological demands during the intrapartum period, where there is repeat distention of vessels and increased tortuosity, can predispose vessels to rupture [4]. In this case presentation, it is indeterminate whether the middle colic pseudoaneurysm was a result from the increased demands associated with pregnancy or there was a pre-existing vascular aberration.

Clinical presentation is variable leading to difficulty in the diagnosis unless there is a high index of suspicion. Symptoms range from vague abdominal discomfort to sudden onset pain and hypovolaemic shock [1,2]. A latent period of several hours may be followed by a rapid progression of symptoms owing to rapidity of extravasation [3]. Radiological investigation should be considered in patients who are not shocked. If appropriate, CT angiogram is considered a sensitive and specific tool in identifying sites of haemorrhage or pseudoaneurysm [1,8]. Notably in the present case, initial CT scanning suggested colitis of the transverse and descending colon, but subsequent deterioration and CT angiography led to the veracious diagnosis.

Due to the severe and abrupt deterioration treatment is thus centered around aggressive resuscitation, surgical exploration, and ligation [3,7]. Any pregnant or postpartum patient with severe abdominal pain and hypovolaemic shock should be evaluated with care and, once common causes for haemoperitoneum are assessed for, consideration should be given to the potential diagnosis of idiopathic spontaneous intraperitoneal haemorrhage [2].

Successful surgical ligation reduces mortality to 8.6 % from 40 to 66 % with non-therapeutic exploratory laparotomy [3,6]. Initial damage control surgery to stabilise, followed by second-look surgery is an accepted approach [7]. In situations where patients are appropriately resuscitated, stabilised and there is evidence of pseudoanerysms on CTA, angiographic embolisation can be considered though on a case to case basis [8].

## 4 Conclusion

Although a rare entity, Idiopathic spontaneous intraperitoneal haemorrhage should be considered when assessing the post-partum patients presenting with unexplained abdominal pain and haemodynamic instability. If clinical status permits, CT angiogram scan provide a sensitive and specific tool diagnosing and identifying sites of haemorrhage or pseudoaneurysm. Management of these patients relies on early recognition, aggressive resuscitation, surgical exploration and ligation.

## Consent

Written informed consent was obtained from the patient for publication of this case report and accompanying images. A copy of the written consent is available for review by the Editor-in-Chief of this journal on request.

## Ethical approval

Ethical approval not applicable.

## Funding

N/A.

This research did not receive any specific grant from funding agencies in the public, commercial, or not-for-profit sectors.

## Author contribution

Dr Danniel Badri – study design, writing the paper.

Dr Callie Killoran – editing the paper.

Dr Ratna Aseervatham – study concept/design, editing the paper.

## Guarantor

Dr Danniel Badri accepts full responsibility for the work and/or the conduct of the study, had access to the data, and controlled the decision to publish.

## Declaration of competing interest

N/A.
